# A polymorphism associated with increased levels of YKL-40 and the risk of early onset of lone atrial fibrillation

**DOI:** 10.1186/1477-5751-12-1

**Published:** 2013-01-02

**Authors:** Kristoffer M A Henningsen, Morten S Olesen, Golnaz Sajadieh, Stig Haunsoe, Jesper H Svendsen

**Affiliations:** 1 Department of Cardiology, The Heart Centre, Rigshospitalet, University of Copenhagen, Copenhagen, Denmark; 2 The Danish National Research Foundation Centre for Cardiac Arrhythmia (DARC), Copenhagen, Denmark; 3 Department of Surgery and Medicine, Faculty of Health Sciences, University of Copenhagen, Copenhagen, Denmark

**Keywords:** YKL-40, Single nucleotide polymorphism, Inflammation, Atrial fibrillation

## Abstract

**Background:**

Plasma levels of YKL-40 are elevated in patients with atrial fibrillation (AF). We hypothesized that a single nucleotide polymorphism (SNP) that affects YKL-40 plasma levels is associated to the risk of lone AF.

**Findings:**

We included 178 young patients with lone AF and the first episode before the age of 40 years, and a control group of 875 healthy individuals. We analyzed a promoter SNP (−131CG) (rs4950928) in the Chitinase 3–like 1 (*CHI3L1*) gene encoding YKL-40, which had previously been associated with elevated levels of YKL-40.

**Conclusions:**

The (−131CG) genotype was not associated with increased risk of AF. Genetically increased YKL-40 levels were not associated to AF.

## Introduction

The chitinase-like protein YKL-40 is emerging as a new biomarker of inflammation, which acts different and independent of C-reactive protein (CRP) [1]. Elevated plasma YKL-40 levels are seen in patients with diseases characterized by inflammation and ongoing tissue remodelling. We have recently shown that YKL-40 is elevated in patients with atrial fibrillation (AF) [2,3].

We hypothesized that a polymorphism in the chitinase-3-like-1 gene (*CHI3L1*), coding for YKL-40 and known to be associated with elevated levels of YKL-40, could be associated with the risk of AF. We focused on patients with early onset *lone* AF as we assumed that the genetic component was relatively larger in these patients compared to older non-*lone* AF patients.

## Methods

A total of 178 patients were included from eight hospitals in the Copenhagen region of Denmark. Patient records from all in- and outpatient activity in the past 10 years with the diagnosis code [ICD-10] I48.9 (Atrial fibrillation and flutter) were identified and read. Only lone AF patients with onset of disease before age 40 years were included.

A control population of 875 healthy volunteers was established [4,5].

ECG and clinical information was collected in order to reduce the possibility of undiagnosed heart disease. All patients and healthy controls were Caucasian.

The study conformed to the principles outlined in the Declaration of Helsinki, and was approved by the local ethics committee of Copenhagen and Frederiksberg. Written informed consent was obtained from patients and healthy volunteers.

We selected a promoter SNP (−131CG) (rs4950928) in the *CHI3L1* gene which had previously been associated with elevated levels of YKL-40 in a GWAS study [6].

SNPs analyses were performed using fluorescence-based real-time PCR (ABI PRISM 7900 Sequence Detection System, Applied Biosystems, CA, USA) and a predeveloped assay (Applied Biosystems).

The genotype distribution was compared between subjects with AF and healthy controls by the Chi-square test (3x2). Statistical analyses were performed using SAS version 9.1 (SAS Institute, Cary, NC, USA).

## Results

The AF patients were significantly younger and significantly more male gender compared to the healthy controls. We found no significant difference in the genotype distributions between the patients with *lone* AF and the healthy controls for the (−131CG) SNP in *CHI3L1* (Table 1). The distribution of the polymorphism for the AF patients and healthy controls were in Hardy-Weinberg equilibrium.

**Table 1:** **Distribution and frequencies of the (131CG) (rs4950928) genotype on *CHI3L1 *for patients with early onset of lone atrial fibrillation and healthy controls**

	**GG**	**GC**	**CC**	**p value**
**AF**	**6(3.3)**	**72(40.5)**	**100(56.2)**	
**Control**	**35(4)**	**321(36.7)**	**519(59.3)**	**ns**

ns: not significant (p>0.05).

## Discussion

This is the first study to examine a variation in the *CHI3L1* gene in relation to AF. The *CHI3L1*gene codes for the YKL-40 glycoprotein. In genome wide association studies (GWAS) SNPs in the promoter region of the *CHI3L1* gene have been associated with higher levels of YKL-40 in plasma [6]. We find no association between the *CHI3L1* (−131CG) genotype and early onset of lone AF.

Elevated plasma concentrations of YKL-40 have previously been associated with diseases in which inflammation is known to play a role in the pathophysiology, such as asthma [7]. We have previously shown that plasma concentrations of YKL-40 are elevated in patients with AF [2,3]. Patients with persistent AF had significantly lower baseline plasma concentration of YKL-40 compared to patients with permanent AF. The results from these studies suggest an association between the inflammatory status and the burden of AF. This study shows that a polymorphism associated with elevated levels of YKL-40 does not represent an increased risk of AF. Unfortunately, we didn’t measure plasma levels of YKL-40, but we speculate that the association between levels of YKL-40 and AF is caused by an activated systemic inflammation leading to YKL-40 elevation, increased fibrosis and to increased risk of AF. Similarly, CRP is known to be robustly associated with increased risk of AF, but genetically elevated CRP is not [8].

## Abbreviations

AF: Atrial fibrillation; SNP: Single nucleotide polymorphism; CHI3L1: Chitinase 3-like 1; CRP: C-reactive protein; GWAS: Genome wide association study; PCR: Polymerase chain reaction.

## Competing interests

The authors declare that they have no competing interests.

## Authors’ contributions

KH, MO and JHS participated in the design of the study. KH and MO carried out the molecular genetic studies, performed the statistical analysis and drafted the manuscript. JHS, GS and SH participated in the coordination of the study and helped to draft the manuscript. All authors read and approved the final manuscript.
